# In-utero exposure to antihypertensive medication and neonatal and child health outcomes: a systematic review

**DOI:** 10.1097/HJH.0000000000001456

**Published:** 2017-07-18

**Authors:** Catherine A. Fitton, Markus F.C. Steiner, Lorna Aucott, Jill P. Pell, Daniel F. Mackay, Michael Fleming, James S. McLay

**Affiliations:** a The Department of Child Health, University of Aberdeen, Royal Aberdeen Children's Hospital, Aberdeen; b Institute of Health and Wellbeing, University of Glasgow, Glasgow, UK

**Keywords:** adverse drug event, antihypertensive agents, child health, drug exposure in pregnancy, female, hypertension, in-utero exposure, pharmacovigilance, preeclampsia, pregnancy-induced hypertension

## Abstract

**Background:**

Although medication is generally avoided wherever possible during pregnancy, pharmacotherapy is required for the treatment of pregnancy associated hypertension, which remains a leading cause of maternal and fetal morbidity and mortality. The long-term effects to the child of in-utero exposure to antihypertensive agents remains largely unknown.

**Objective:**

The aim of this study was to systematically review published studies on adverse outcomes to the child associated with in-utero exposure to antihypertensive medications.

**Methods:**

OVID, Scopus, EBSCO Collections, the Cochrane Library, and Web of Science databases were searched for relevant publications published between January 1950 and October 2016 and a total of 688 potentially eligible studies were identified.

**Results:**

Following review, 47 primary studies were eligible for inclusion. The Critical Appraisal Skills Programme checklist was used to assess study quality. Five studies were of excellent quality; the remainder were either mediocre or poor. Increased risk of low birth weight, low size for gestational age, preterm birth, and congenital defects following in-utero exposure to all antihypertensive agents were identified. Two studies reported an increased risk of attention deficit hyperactivity disorder following exposure to labetalol, and an increased risk of sleep disorders following exposure to methyldopa and clonidine.

**Conclusion:**

The current systematic review demonstrates a paucity of relevant published high-quality studies. A small number of studies suggest possible increased risk of adverse child health outcomes; however, most published studies have methodological weaknesses and/or lacked statistical power thus preventing any firm conclusions being drawn.

## INTRODUCTION

Hypertensive disorders of pregnancy have been identified as the leading cause of maternal death in industrialized countries, complicating approximately 7% of pregnancies [1,2].

Prior to pregnancy, approximately 3% of women have an existing diagnosis of hypertension of whom a quarter will go on to develop preeclampsia [2,3]. A further 4% will develop hypertension during the course of their pregnancy and also be at higher risk of developing preeclampsia [4].

Consequently, there has been a move to treat hypertensive women of childbearing age more aggressively in an attempt to normalize blood pressure (BP) prior to and during pregnancy and reduce the occurrence of preeclampsia [5]. Despite hypertensive disorders of pregnancy being a common problem and suggestions that hypertension during pregnancy may influence the developing fetus and child, the efficacy of antihypertensive agents in terms of maternal and fetal outcomes and the possible long-term effects of in-utero antihypertensive exposure on the developing fetus and child remain largely unknown [6].

Although in-utero exposure to any medication during the first trimester is associated with the highest risk of teratogenic malformations, exposure during the second and third trimesters has also been linked to functional and behavioural abnormalities, which may not be immediately apparent [7–9].

Although the fetogenic and teratogenic risks associated with angiotensin-converting enzyme inhibitors (ACEi) and AT1 blockers (ARB) use during pregnancy are well recognized [4] the potential effects of methyldopa, beta-blockers, such as labetalol and the calcium channel blocker nifedipine, are less clear [9,10]. In-utero exposure to these agents has been associated with low birth weight, childhood depression, delayed neurocognitive development [11,12], increased incidence of childhood asthma [12,13], and neonatal seizures and hematological disorders [14,15]. However, the data from any one study are not sufficiently robust to make meaningful comment on the strength of these associations [13,16].

To determine whether in-utero exposure to antihypertensive medication is associated with adverse child outcomes, we reviewed primary literature reporting child outcomes following exposure *in utero* to one or more antihypertensive medications compared with unexposed children.

## METHODS

### Search strategy

A systematic review protocol was designed as per standard Preferred Reporting Items for Systematic Reviews and Meta-Analyses (PRISMA) guidelines [17]. The databases Medline, Cumulative Index to Nursing and Allied Health Literature (CINAHL), Cochrane Database of Systematic Review library, Scopus (Elsevier), and EBSCO (psycINFO, CINAHL Plus) were searched for publications from January 1950 to October 2016 inclusive (last search 10 October 2016), using the search terms (contained in keywords, title, or abstract) of prenatal, antenatal, calcium channel blocker, beta blocker, ACEi, adrenergic receptor antagonist/blocker, alpha-2 adrenergic receptor agonist, angiotensin II receptor, antihypertensive, hydralazine, sodium nitroprusside, clonidine hydrochloride, moxonidine, renin inhibitor, thiazide, loop diuretic, and potassium sparing diuretic. Ineligible articles were excluded using the following search terms (contained in keywords, title, or abstract) animal, rat, mice, haemanginoma∗, mouse, Doppler, ultrasound, ambulatory, willingness to pay, adherence, transplant, herbal, tumor, imaging, HIV, mercury, vitamin, and gene∗. Wildcard symbols, truncation, combinations of search terms using Boolean operators, and alterative spellings were used. Included studies reported pregnant female patients of any ethnicity; exposed to one or more agents of interest (beta-adrenoceptor blocking drugs; vasodilator antihypertensive drugs; centrally acting antihypertensive drugs; alpha-adrenoceptor blocking drugs; ACEi; angiotensin II receptor antagonists; renin inhibitors; calcium channel blockers; thiazide diuretics; loop diuretics; potassium sparing diuretics) administered via any route compared with no drug, placebo or any active antihypertensive comparator. There was no restriction on the type of hypertension being treated, that is, preexisting hypertension, gestational hypertension, and preeclampsia. Eligible studies evaluated any health outcomes in offspring, as measured using well defined diagnostic criteria and doctor diagnoses. Studies reporting the results of controlled trials, case–control studies, cohort studies, and meta-analysis with synthesized data were included.

The reference lists from eligible papers were scanned for other relevant studies. The search was limited to English and German language articles. Narrative and systematic reviews (with no synthesis of data), studies published only as abstracts, letters, or conference proceedings, discussion papers, animal studies, and editorials were excluded.

Initial screening of titles was carried out to identify potentially relevant studies, followed by screening of abstracts and then by full paper review. All titles and abstracts were independently evaluated by two reviewers (M.S. and J.S.M.) for consistency of inclusion/exclusion.

### Study review and data extraction

Quality assessments were conducted by two independent reviewers (C.F. and M.S.) using a modified version of the Critical Appraisal Skills Programme quality assessment tool for randomized controlled trials [18], case–control studies [19], and cohort studies [20]. Data extracted for randomized controlled trials were: the generation of allocation sequence, concealment of allocation, outcome measures, and other risks of bias, and for case–control and cohort studies, appropriateness of case and control recruitment, inclusion and exclusion criteria clearly stated, appropriate validation of cases, appropriate analyses, and sufficient follow-up information. Where information was missing from the studies, contact with authors was attempted, where practical, via e-mail; however, no responses were received. Due to lack of study homogeneity, meta-analysis was not appropriate. Therefore, a narrative synthesis of the results was conducted.

### Registration and reporting

The current review was registered with the International Prospective Register of Systematic Reviews: http://www.crd.york.ac.uk/prospero/[21]. The PRISMA checklist was used to guide the reporting of the systematic review: http://www.prisma-statement.org/.

## RESULTS

The database searches retrieved 816 citations. Removal of duplicates followed by review of titles and abstracts yielded 47 relevant studies. The PRISMA flowchart (Supplemental file: Fig. 1) illustrates the number of titles, abstracts, and full papers excluded.

In total, 22 studies were conducted in Europe; 16 in North America, three in Asia, one in central America, and one in Africa. Four of the 47 studies were multinational, three of which were across continents [22–25]. Of these 47 studies, 29 were published after 2000. The reported study populations ranged from 22 to 2529 636 participants (Tables 1–4). Seven studies reported on all classes of antihypertensive medication [26–32], five reported on calcium channel blockers only [23,33–36], 12 on beta-blockers only [37–48], two on methyldopa only [49,50], six on methyldopa and/or beta-blockers [11,13,51–54], two on calcium channel blockers and/or beta blockers [12,55], nine on ARBs or ACEis [22,28,29,56–60], one on clonidine only [61], one diuretics only [25], one compared nifedipine with prazosin [62], one compared nifedipine with hydralazine [63], and one compared labetalol with hydralazine [64]. Reported outcomes were predominantly the rate of malformations and birth outcomes (i.e., perinatal death, birthweight, size for gestational age, and preterm birth), but four publications also included childhood assessments at later follow-up such as intelligence quotient (IQ) and behavioural outcomes [11,13,49,61]. Results were reported as a mixture of crude odds ratios (ORs), adjusted ORs (adjusted OR) or relative risks (RRs).

Descriptive tables (Tables 1–6) showing drugs and outcomes investigated were produced. Results where outcomes were found to be significant in some way are marked with Y, and those not found to be significant marked with *N*.

Studies were categorized according to study design and quality and bias assessed accordingly (Supplemental files: Tables 7–9). No study achieved the maximum score for quality, five were graded as excellent [29,31,33,51] and 26 as fair [11–13,25–28,30,32,35,40,42–45,47,49,50,52–54,60,62–64]. The remainder [16] were graded as poor quality primarily due to a lack of appropriate controls, and/or documented methods for selection of patients and controls for inclusion [22–24,36–39,41,46,48,55,56,58,59,61,65].

For clarity and ease of interpretation, the results of the eligible studies have been reported, according to the drug of exposure, as early (birth to 1 month) and long-term (>1 month) outcomes.

### Studies assessing early outcomes following any antihypertensive exposure

Five case–control studies [26–28,30,31] and two cohort studies [29,32] reported the effects of in-utero exposure, on the fetus and child, of any antihypertensive agent. Table 1 reports study type, outcomes, and number of participants for studies assessing in-utero exposure to any antihypertensive medication. Two studies were graded as excellent [29,31] and the other five studies [26–28,30,32] as mediocre in quality.

In the cohort study of 1418 women, rated as excellent, Lennestål *et al.*[29] reported that offspring exposed to antihypertensive medication during pregnancy were more likely to be born preterm [adjusted OR 3.3; 95% confidence interval (CI) 2.9–3.8], small for gestational age (adjusted OR 4.2; 95% CI 3.6–5.0) and be hypoglycemic (adjusted OR 2.6; 95% CI 2.0–3.4) compared with offspring not exposed to antihypertensive therapy during pregnancy. Lennestål *et al.*[29] also reported an increased odds of any congenital malformation (adjusted OR 1.5; 95% CI 1.2–1.9); particularly cardiovascular defects (adjusted OR 2.6; 95% CI 1.9–3.5) and increased risk of septal defects (RR 2.7; 95% CI 1.7–4.0). There was an increased risk of congenital malformation with exposure to two or more different groups of antihypertensive patients (RR 4.0; 95% CI 1.8–7.6), compared with unexposed women. However, no increased risk of hypospadias (RR 1.3; 95% CI 0.5–2.9) and central nervous system malformations (RR 2.2; 95% CI 0.7–5.1) was reported in offspring following in-utero exposure to any antihypertensive agent.

In the case–control study rated as excellent, studying major congenital anomalies (4155 cases, 54 878 controls), and being small for gestational age (7445 cases, 48 889 controls), Nakhai-Pour *et al.*[31] reported increased odds of being small for gestational age when mothers were exposed to antihypertensive medication during the second or third trimester of pregnancy, compared with no antihypertensive exposure (adjusted OR 1.5; 95% CI 1.2–2.0). There was no increased odds of major congenital anomalies following exposure to antihypertensive medication during pregnancy in any trimester, compared with no antihypertensive exposure.

Banhidy *et al.*[26] reported the immediate outcomes of pregnancy and efficacy of in-utero exposure to antihypertensive medication among 37 734 offspring, in a case–control study rated as mediocre. Both chronic and gestational hypertensions were assessed. Antihypertensive treatment of mothers with chronic hypertension was associated with an increased risk of preterm birth (adjusted OR 1.5; 95% CI 1.3–1.8) compared with the untreated hypertensive group. No significant increase was observed for mothers treated for gestational hypertension (adjusted OR 1.2; 95% CI 0.9–1.5), compared with the untreated hypertensive reference group. The authors also reported that mothers with chronic hypertension, who were treated with antihypertensive medication, had significantly lighter babies (*P* < 0.0001) and increased odds of low birth weight (adjusted OR 2.3; 95% CI 1.8–2.7) [26]. However, there was no significant difference in low birth weight offspring between mothers treated for gestational hypertension (adjusted OR 1.5; 95% CI 1.0–2.1) and the untreated hypertensive group [26].

Caton *et al.*[27] reported the exposure to in-utero antihypertensive medication in a mediocre case–control study of 758 offspring with hypospadias vs. 2058 offspring without hypospadias. Early antihypertensive use (1 month preconception to 4 months postconception) was not associated with increased odds of hypospadias (adjusted OR 1.4; 95% CI 0.7–2.9), although, late use of antihypertensive medication (4 months postconception onwards) was (adjusted OR 5.0; 95% CI 1.9–12.9). However, untreated hypertension during pregnancy was also associated with increased odds of hypospadias (adjusted OR 2.1; 95% CI 1.6–2.9).

In a second case–control study rated as mediocre, by Caton *et al.*[28], 5021 cases with a cardiovascular malformation and 4796 controls were assessed and the prevalence of hypertension and antihypertensive use during pregnancy was reported. Exposure to antihypertensive medication during the first trimester was associated with increased odds of pulmonary valve stenosis (OR 2.6; 95% CI 1.3–5.4), Ebstein malformation (OR 11.4; 95% CI 2.8–34.1), coarctation of the aorta (OR 3.0; 95% CI 1.3–6.6), and secundum atrial septal defects (OR 2.4; 95% CI 1.3–4.4). Antihypertensive treatment initiated after the first trimester was also associated with increased odds of pulmonary valve stenosis (OR 2.4; 95% CI 1.1–5.4), perimembranous ventricular septal defects (OR 2.3; 95% CI 1.2–4.6), and secundum atrial septal defects (OR 2.4; 95% CI 1.3–4.4). There were no increased odds associated with untreated hypertension for any of the cardiovascular malformations investigated.

Van Gelder *et al.*[30] reported increased odds of hypospadias associated with treatment of chronic hypertension in the early stages of pregnancy (adjusted OR 2.9; 95% CI 1.1–7.4) in a case–control study, rated as mediocre, assessing a total of 5568 cases and 7253 controls. For mothers with gestational hypertension, late initiation of antihypertensive treatment was associated with increased odds of ventricular septal defects (adjusted OR 2.7; 95% CI 1.1–6.8), and left-sided cardiac defects (adjusted OR 4.3; 95% CI 1.5–12.3) in offspring. Preeclampsia superimposed upon chronic hypertension, with early use of antihypertensive medication, was associated with increased odds of ventricular septal defects (adjusted OR 3.7, 95% CI 1.3–10.7), left-sided cardiac defects (adjusted OR 3.8; 95% CI 1.1–13.2), and cleft lip/palate (adjusted OR 5.0; 95% CI 1.3–19.0) in offspring. However, untreated preeclampsia superimposed upon chronic hypertension was also associated with cardiovascular malformations (adjusted OR 3.7; 95% CI 1.4–9.7), ventricular (adjusted OR 3.9; 95% CI 1.3–11.7), and atrial septal defects (adjusted OR 6.5; 95% CI 1.8–23.7) when compared with the untreated normotensive control group. Untreated preeclampsia was also associated with ventricular septal defects (adjusted OR 1.5; 95% CI 1.1–2.2), and hypospadias (adjusted OR 2.4; 95% CI 1.4–4.0) compared with the control group. There were no increased odds of congenital malformations associated with untreated gestational hypertension in comparison with the control group. In a mediocre cohort study of 59 mildly hypertensive women, Arias *et al.*[32] reported no significant differences in gestational age, fetal weight, proportion of low birth weight babies, Apgar scores at 1 and 5 min, or intrauterine growth restriction, between hypertensive women treated with antihypertensive patients, and untreated hypertensive women.

*Any hypertensive summary.* In summary, although seven studies reported increased odds of preterm birth and low birth weight in treated mothers, particularly those with chronic hypertension and four studies, a possible increase in the incidence of congenital malformations study designs did not allow assessment of the relative importance of hypertension vs. exposure to antihypertensive patients. The ORs for perinatal mortality, preterm birth, and congenital cardiovascular defects are reported as Forest plots in Supplementary Figs. 2–4.

### Beta-blocker exposure

One case–control study [28], 16 cohort studies [11,12,29,37–41,43,45,46,48,51,55,60,65], and seven randomized controlled trials (RCTs) [42,44,47,52–54,64] have reported the effects of in-utero exposure to beta-blockers, on the fetus and child. Table 2 reports details on study type, outcomes, and number of participants for studies assessing in-utero exposure to beta-blocker medication. Eight studies were poor [37–39,41,46,48,55,65], and 14 were of mediocre quality [11,12,28,40,42–45,47,52–54,60,64] with two studies being of excellent quality [29,51].

In a cohort study of 1063 238 pregnant women, rated as excellent, Lennestål *et al.*[29] reported increased odds of cardiovascular malformations (adjusted OR 2.8; 95% CI 1.8–4.1) in beta-blocker-treated women, compared with those who did not report antihypertensive use during pregnancy. The authors [29] also reported 19 deaths among the 1444 offspring exposed to any antihypertensive medication (RR 1.9; 95% CI 1.0–3.0); 15 of whom had been exposed to a beta-blocker in either early or late pregnancy.

In a cohort study of 100 029 women, also rated as excellent, Orbach *et al.*[51] reported increased odds of preterm birth among the 107 women taking atenolol (adjusted OR 2.7; 95% CI 1.6–4.6), in comparison with a normotensive untreated reference group. The authors [51] also reported increased odds of being small for gestational age (adjusted OR 4.8; 95% CI 2.1–11.1), low birth weight (adjusted OR 3.9; 95% CI 2.4–6.3), and intrauterine growth restriction (adjusted OR 5.2; 95% CI 2.6–10.4) in offspring exposed in-utero to atenolol during the third trimester, compared with the normotensive untreated reference group.

However, there was also increased odds of preterm birth (adjusted OR 1.9; 95% CI 1.6–2.3), being small for gestational age (adjusted OR 2.1; 95% CI 1.4–3.0), and intrauterine growth restriction (adjusted OR 2.1; 95% CI 1.5–2.9), for offspring exposed to untreated maternal hypertension in-utero compared with the normotensive untreated reference group. The authors [51] did not report any significant association with congenital defects (adjusted OR 1.3; 95% CI, 0.8–2.0) and no perinatal deaths occurred.

In a mediocre cohort of 911 685 women, Meidahl Petersen *et al.*[40], reported increased odds of preterm birth in the 2259 women taking any beta-blocker (OR 2.3; 95% CI 2.0–2.5) compared with untreated normotensive women. The authors [40] also reported increased odds of being small for gestational age (adjusted OR 2.0; 95% CI 1.7–2.4) and perinatal mortality (adjusted OR 1.9; 95% CI 1.3–2.9) following exposure to beta-blockers in offspring, compared with an untreated reference group.

Ray *et al.*[43], in a study of 1948 women rated as mediocre, reported increased odds of preterm birth (adjusted OR 5.1, 95% CI 3.8–6.9) and being small for gestational age (adjusted OR 2.3; 95% CI 1.6–3.3) but no significantly increase of perinatal mortality (adjusted OR 1.5; 95% CI 0.6–3.4) following in-utero exposure to beta-blocker, in comparison with an untreated hypertensive comparison group.

In a mediocre cohort study of 312 women, Lydakis *et al.*[45] reported an increased risk of preterm birth (33.0 vs. 15.4%; *P* < 0.001), low birth weight (2.4 vs. 3.1 kg; *P* < 0.05), and being small for gestational age (48.7 vs. 20.9%; *P* < 0.001) following atenolol exposure compared with an untreated hypertensive comparison group, with the largest risk of being small for gestational age following exposure to atenolol during early pregnancy (70, 30, and 39% for treatment started less than 20, 20–30, and more than 30-week gestation, respectively; *P* = 0.01).

In a cohort study of 1223 women rated as poor, Xie *et al.*[65] reported increased odds of infant hospitalization for respiratory distress syndrome, sepsis, and seizures in those who were exposed to labetalol during pregnancy, compared with those who were exposed to methyldopa (adjusted OR 1.5; 95% CI 1.0–2.2). The authors, however, did not report increased odds of being born preterm (adjusted OR 1.0, 95% CI 0.9–1.2), or perinatal mortality (adjusted OR 1.0; 95% CI 0.3–3.0).

In a cohort study rated as poor, investigating the possible effects of labetalol for the treatment of severe preeclampsia, Heida *et al.*[38] reported no increased risk of low birth weight (1.5 vs. 1.6 kg; *P* = 0.25), being small for gestational age (21.8 vs. 22.2%; *P* = 0.96), or being born preterm (89.1 vs. 79.6%; *P* = 0.17), in 109 offspring, 55 of whom were exposed *in utero* to labetalol [38]. The authors did however report an increased risk of perinatal mortality (4.6 vs. 0%; *P* = 0.02) associated with labetalol. In a poor cohort study of 491 women, Bayliss *et al.*[37] reported an increased risk of being small for gestational age (70 vs. 21 or 26%), lower birth weight (mean 2.2 vs. 3.1 kg; *P* < 0.01), and lower ponderal index (22.3 vs. 22.6 or 24.5 kg/m^3^ × 10^4^; *P* < 0.01) following in-utero exposure to atenolol in the first trimester of pregnancy, compared with untreated hypertensive women.

Lip *et al.*[39] reported significantly lower mean birth weight in offspring exposed to atenolol compared with babies exposed to other beta-blockers or methyldopa, or born to mothers with untreated hypertension (mean 2.2, 2.3, 2.7, and 2.8 kg, respectively; *P* < 0.001) in a cohort study, rated as poor, of 398 women. The authors [39] also reported a lower ponderal index, following in-utero exposure to atenolol, in comparison with the untreated hypertensive group (2.2 vs. 2.4 kg/m^3^ × 10^4^; *P* = 0.001).

Caton *et al.*[28], in a mediocre case–control study of 9817 offspring, reported increased odds of cardiovascular malformations (adjusted OR 2.6; 95% CI 1.9–3.5) and pulmonary valve stenosis (adjusted OR 5.0; 95% CI 1.8–13.8), following in-utero beta-blocker exposure, compared with normotensive controls. In a mediocre cohort study of 87 407 offspring, Davis *et al.*[12] reported that exposure to beta-blockers during the third trimester was associated with an increased risk of hypoglycemia (RR 3.1; 95% CI 2.2–4.2), and feeding problems (RR 1.8; 95% CI 1.3–2.5), in comparison with the normotensive untreated reference group; however, the authors did not report any increased odds of any congenital defect (OR 1.0; 95% CI 0.8–1.2) or cardiac defects (OR 1.9; 95% CI 0.7–5.2)

In a mediocre RCT of 100 women assessing fetal head circumference and placental weight, Fidler *et al.*[52] observed no significant differences between the effects of in-utero exposure to oxoprenolol and those exposed to methyldopa or the untreated control group. Rubin *et al.*[44] reported the results of a mediocre RCT investigating the in-utero effects of atenolol vs. placebo in 120 women with mild-to-moderate pregnancy associated hypertension. The authors reported no difference in intrauterine growth retardation, Apgar score, incidence of hypoglycemia or hyperbilirubinemia, or neonatal BP between the two treatment groups. However, they did report increased occurrence of neonatal bradycardia in the atenolol exposed group (*P* < 0.01). In a poor cohort study investigating the effects of labetalol on 129 offspring, Munshi *et al.*[41] reported a significantly increased incidence of hypoglycemia in offspring exposed to labetalol, compared with the reference group who were exposed to other antihypertensive agents (*P* < 0.01); however, there was no significant difference in the incidence of intrauterine growth retardation or birth asphyxia.

In a poor cohort study of 22 women, Macpherson *et al.*[48] reported pregnancy outcomes of labetalol exposure during pregnancy in hypertensive women (*N* = 11), compared with a comparison group of unexposed, normotensive women (*N* = 11). There was no difference in heart rate (HR), respiratory rate or palmar sweating between the two treatment groups; however, mean SBP in infants was significantly different at 2-h post birth (*P* < 0.05). This difference disappeared after 72 h.

In an RCT of 176 women rated as mediocre, Plouin *et al.*[53] reported no difference in HR, BP, respiratory rate or glucose levels between labetalol and methyldopa exposed offspring. In a mediocre RCT of 200 women with mild preeclampsia, Sibai *et al.*[47] reported no significant difference in birth weight and neonatal ICU (NICU) admission between a group treated with labetalol and hospitalization and a group treated with hospitalization only. There was a significant difference in offspring being small for gestational age (19.1% with labetalol and hospitalization vs. 9.3% with hospitalization only; *P* < 0.05).

Lieberman *et al.*[46] reported the results of a cohort study, rated as poor, which involved eight hypertensive women who took propranolol with or without other antihypertensive patients, and a comparison group of 15 hypertensive women who took antihypertensive patients, but did not take propranolol. A significantly higher proportion of offspring in the propranolol exposed group were below the 5th percentile for birthweight compared with the comparison group (propranolol 7/9 vs. other antihypertensive patients 4/15; *P* = 0.02).

In a mediocre RCT of 263 women with mild-to-moderate chronic hypertension, Sibai *et al.*[54] reported no significant differences in preterm delivery, being small for gestational age, 1 and 5 min Apgar scores, gestational age, birthweight, congenital abnormalities, fetal hypotension, fetal bradycardia, and head circumference between the control group and both the labetalol-treated and methyldopa-treated groups. In a RCT of 200 women (100 given labetalol, 100 given hydralazine) rated as mediocre, Vigil-De Gracia *et al.*[64] reported a significant difference in offspring with neonatal bradycardia (10.6% labetalol vs. 1.9% hydralazine; *P* = 0.008) and hypotension (10.6% labetalol vs. 3.9% hydralazine; *P* = 0.05). No differences in birthweight, intrauterine growth restriction, hypoglycemia, respiratory distress syndrome, 1 and 5 min Apgar scores, or NICU admissions were reported between the two treatment groups.

In a poor cohort study to investigate functional development of the fetal central nervous system, involving 117 women, 21 of whom were treated with nifedipine or labetalol for pregnancy-induced hypertension, Gazzolo *et al.*[55] reported disturbances in the development of in-utero fetal behavioral recordings in the treated cohort. However, when the authors matched exposed and unexposed cases for age and birth weight following delivery, they concluded that impairment of the developing state was due to confounding rather than antihypertensive exposure.

*Beta-blocker summary*: In summary, exposure to beta-blockers in the setting of hypertension, was associated with an increased risk of preterm birth, being small for gestational age, increased perinatal mortality and low birth weight; however, there was limited evidence to suggest that this increased risk was due to the medication rather than the underlying hypertensive disease, with only six of 13 studies reporting increased risk controlling for underlying hypertension. There was also some evidence of an increased risk of cardiovascular malformations; however, relevant studies did not include an untreated comparison group [28,29]. Three RCTs [42,44,53], which included appropriate control groups, reported no significant risk of other outcomes, including hypotension, depressed respiratory rate, and hypoglycemia. The ORs for perinatal mortality, preterm birth, and congenital cardiovascular defects are reported as Forest plots in Supplementary Figs. 2–4.

### Calcium channel blocker exposure

Three RCTS [34,62,63], one case–control study [35] and four cohort studies [12,23,24,33] have reported the effects of in-utero exposure to calcium channel blockers on the fetus and child. Table 3 reports details on study type, outcomes, and number of participants for studies assessing in-utero exposure to calcium channel blocker medication. One study was graded as excellent quality [33], five as mediocre [12,34,35,62,63] and the remaining three studies as poor in quality [23,24,36].

In the one study rated as excellent, Bateman *et al.*[33] reported no increased odds of neonatal seizures associated with calcium channel blocker exposure, compared with those unexposed to any calcium channel blocker in the third trimester of pregnancy (adjusted OR 1.0; 95% CI 0.7–1.3) in a cohort of 2529 636 women.

Gruppo [34], in an RCT of 283 women, rated as mediocre, reported no increased risk of low birthweight, spontaneous abortion, intrauterine death, or admission to NICU after birth. Questionnaire follow-up at 18-month postdelivery of 190 of the 283 women [36], demonstrated no increased risk of congenital malformations, or in occurrence of health problems (impairments in gross/fine motor skills, hearing, sight or language, or respiratory conditions) between offspring exposed *in utero* to nifedipine and offspring without in-utero antihypertensive exposure.

Magee *et al.*[23] reported an increased rate of preterm birth following in-utero calcium channel blocker exposure (28 vs. 9%; *P* = 0.003) compared with normotensive untreated women in a poor cohort study of 156 women. The authors [23] reported no increased risk of low birth weight (mean 3.0 kg vs. 3.4 g; *P* = 0.08), or congenital malformations (3 vs. 0%; *P* = 0.27) among offspring with in-utero exposure to calcium channel blockers.

Weber-Schoendorfer *et al.*[24], in a cohort study of 1105 women rated as poor, reported increased odds of preterm birth (adjusted OR 4.6; 95% CI 2.9–7.3), low mean birth weight (3.2 vs. 3.3 kg; *P* = 0.007), miscarriages (14 vs. 7.6%; adjusted OR 2.2; 95% CI 1.4–3.5) associated with in-utero exposure to calcium channel blockers compared with an untreated normotensive group. The authors [24] reported no significantly increased odds of stillbirths (adjusted OR 3.0; 95% CI 1.0–8.7), or birth defects (3.5 vs. 1.9%; *P* = 0.10) with in-utero exposure to calcium channel blockers compared with an untreated normotensive group.

Hall *et al.*[62] reported the results of a mediocre RCT assessing the addition of nifedipine or prazosin to existing antihypertensive medication in 145 women. The use of prazosin was associated with a higher number of intrauterine deaths compared with nifedipine (7 vs. 1; *P* = 0.03).

In a mediocre case–control study of 54 016 women, involving 22 865 cases taken from the Hungarian Congenital Abnormality Registry and 38 151 controls without malformations, Sorensen *et al.*[35] reported increased odds of hypospadias (adjusted OR 3.5; 95% CI 1.4–8.6) following in-utero calcium channel blocker exposure during the first trimester, compared with the control group. Exposure to calcium channel blockers between 4 and 9 months of gestation was also associated with increased odds of cardiovascular abnormalities (adjusted OR 1.4; 95% CI 1.2–1.7), undescended testis (adjusted OR 1.5; 95% CI 1.1–1.9), and multiple congenital abnormalities (adjusted OR 1.4; 95% CI 1.0–1.9) in cases compared with controls.

In an RCT of 49 hypertensive women rated as mediocre, Fenakel *et al.*[63] reported no significant difference in birth weight, gestational age, congenital malformations, and perinatal death between the offspring of 24 hypertensive women treated with nifedipine and 25 hypertensive women treated with hydralazine.

In a mediocre cohort study of 87 407 pregnant women, Davis *et al.*[12] reported an increased risk of congenital abnormalities of the upper alimentary tract (RR 7.2; 95% CI 1.9–27.5) in offspring exposed to calcium channel blockers during the first trimester in comparison with an untreated normotensive reference group. Davis *et al.*[12] also reported an increased risk of perinatal jaundice (RR 1.4; 95% CI 1.2–1.6), hematological disorders (RR 2.6; 95% CI 1.4–5.1) and convulsions in the new-born (RR 3.6; 95% CI 1.3–10.4) following in-utero exposure during the third trimester of pregnancy.

*Calcium channel blocker summary*. In summary, the results of the calcium channel blocker studies are mixed, with evidence of increased perinatal mortality from two RCTs, increased odds of preterm birth and perinatal mortality from the cohort studies, and evidence of increased malformations from the case–control study. None of the reviewed studies assessed the effect of untreated hypertension on outcomes, thus limiting the interpretation of the data and the relative importance of in-utero exposure rather than underlying disease. The ORs for perinatal mortality, preterm birth, and congenital cardiovascular defects are reported as Forest plots in Supplementary Figs. 2–4.

### Angiotensin-converting enzyme inhibitor and AT1 blocker exposure

One case–control study [28] and seven cohort studies [22,29,56–60] have reported the effects of in-utero exposure to ACEis and ARBs on the fetus and child. Table 4 reports details on the study types, outcomes, and number of participants for studies assessing in-utero exposure to ACEi or ARB medication. Four studies were of poor quality [22,56,58,59], two were of mediocre quality [28,60], and two were excellent quality [29,57].

In a cohort study involving 465 754 offspring, rated as excellent, Li *et al.*[57] reported increased odds of any birth defect with exposure to ACEi therapy during the second or third trimester compared with an untreated normotensive group (adjusted OR 2.3; 95% CI 1.1–5.2). There were no increased odds of congenital defects (adjusted OR 1.9; 95% OR 0.8–4.2) associated with in-utero ACEi exposure in comparison with an untreated hypertensive group. The authors [57] reported no increased risk of congenital defects in the first trimester following in-utero ACEi exposure compared with both an untreated normotensive group and an untreated hypertensive group. The authors did report increased odds of any congenital defect among the offspring of untreated hypertensive women, in comparison with those of untreated normotensive women (OR 1.25; 95% CI 1.2–1.3).

In a cohort study involving 1063 238 women, rated as excellent, Lennestål *et al.*[29] reported no significantly increased risk of congenital malformations (3.3%; RR 2.9; 95% CI 0.9–6.8) associated with the use of ACEis during pregnancy compared with those who did not report any antihypertensive use. There was, however, an increased risk with exposure to any antihypertensive excluding ACEis (OR 2.54; 95% CI 1.84–3.52) in comparison with no antihypertensive use.

In contrast, in a cohort study rated as poor, comparing ACEi and/or ARB (252 women) therapy vs. other antihypertensive therapy (256 women) or nonteratogenic exposure (495 women), Diav-Citrin *et al.*[22] reported an increased incidence of preterm delivery (21.2 vs. 8.0%; *P* < 0.001), low birth weight (median 3.0 vs. 3.3 kg; *P* < 0.001), lower gestational age (median 38 vs. 40 weeks; *P* < 0.001), and miscarriage (11.5 vs. 6.3%; *P* = 0.021) associated with in-utero exposure to an ACEi or ARB, in comparison with a nonteratogen-exposed group. Diav-Citrin *et al.*[22] did not observe an increased rate of stillbirth (1.2 vs. 0.8%; *P* = 0.838) or congenital malformations (4.2 vs. 3.8%; *P* = 0.954).

Tabacova *et al.*[59] reported a stillbirth rate of 11.0%, a preterm delivery rate of 64.3%, and a congenital abnormality rate of 32.5%, following enalapril exposure in 108 pregnancies, in a poor cohort study; however, there was no comparison group. The authors [59] also reported oligohydramnios and specific adverse outcomes (limb deformities, cranial ossification deficits, lung hypoplasia), thought to be secondary to reduced amniotic fluid volume rather than neonatal renal failure.

Moretti *et al.*[58] reported an increased incidence of miscarriage (*P* < 0.001), lower birth weight (*P* < 0.001), and lower gestation ages (*P* < 0.001) in those exposed to ACEi or ARBs compared with those exposed to other antihypertensive medications and untreated normotensive mothers, in a cohort study of 388 women, rated as poor. The authors [58] reported no increased risk of congenital malformations following in-utero ACEi or ARB exposure in comparison with other antihypertensive patients or the untreated normotensive reference (1.8, 1.9, and 1.6%, respectively; *P* = 0.99).

Caton *et al.*[28] reported increased odds of being prenatally exposed to antihypertensive ACEi/ARBs in cases with Ebstein malformation (OR 26.4; 95% CI 2.3–306) in a mediocre case–control study of 5021 cases and 4796 controls. Karthikeyan *et al.*[56] reported a similar miscarriage rate (11.8 vs. 10.0%), and developmental abnormality rate (8.8 vs. 10.0%) between ACEis and ARBs, in a poor cohort study of 94 women. In a mediocre cohort study involving 29 507 offspring, Cooper *et al.*[60] reported an increased risk of malformations associated with exposure to ACEis in the first trimester (7.1 vs. 2.6%; RR 2.7; 95% CI 1.7–4.3).

*ACEi and ARB summary.* In summary, ACEi and ARBs were associated with an increased risk of preterm delivery and miscarriage. The results relating to congenital abnormalities were conflicting with four studies reporting no increased risk and five reporting increased risk. The results, as a whole, are further confounded by the lack of inclusion of an untreated hypertensive group. The ORs for congenital cardiovascular defects are reported as a Forest plot in Supplementary Fig. 4.

### Methyldopa exposure

Two cohort studies [51,65] and three RCTs [49,50,52] reported the results of in-utero exposure to methyldopa and outcomes in the fetus and child. Table 5 reports details on the study type, outcomes, and number of participants for studies assessing in-utero exposure to methyldopa. One study was of poor quality [65], three were of mediocre quality [49,50,52], and one was scored as excellent [51] on both the quality and bias tools.

In the only study rated as excellent, Orbach *et al.*[51] reported an increased incidence of preterm birth (adjusted OR 4.2; 95% CI 3.2–5.5), perinatal death (adjusted OR 2.1; 95% CI 1.1–4.0), low birth weight (adjusted OR 3.8; 95% CI 2.9–4.9), intrauterine growth restriction (adjusted OR 4.3; 95% CI 2.8–6.6), and low Apgar scores (≤7) at 1 min (adjusted OR 2.0; 1.4–2.7) and 5 min (adjusted OR 2.8; 95% CI 1.5–5.4), following exposure to methyldopa in the third trimester in 99 514 women, in comparison with an untreated normotensive reference group.

In a cohort study of 1223 women rated as poor, Xie *et al.*[65] reported similar preterm birth rates (27.3 vs. 26%) in those exposed to methyldopa during pregnancy, compared with those who were exposed to labetalol. Cockburn *et al.*[49] reported no differences in BP, or health issues between babies exposed in-utero to methyldopa, and those unexposed in a mediocre RCT of 195 women. At 4 years of age, boys in the unexposed group has larger head circumferences than those exposed to methyldopa between 16 and 20-week gestation (*P* < 0.05). However, there was no difference in IQ or behavior.

Fidler *et al.*[52] observed no difference in head circumference, placental weight, or adjusted birth weight between the methyldopa exposed group and an oxoprenolol or nonexposed group in a mediocre RCT of 100 women. In a RCT of 25 women, Weitz *et al.*[50] reported no significant differences in birthweight, head circumference, ponderal index, perinatal death, or 1 and 5 min Apgar scores between 13 hypertensive mothers treated with methyldopa during pregnancy and 12 hypertensive mothers given a placebo, in a mediocre RCT.

*Methyldopa summary.* In summary, methyldopa exposure was associated with an increased risk of preterm birth, perinatal mortality, and low birth weight. The RCTs reported no difference among methyldopa exposed offspring in other outcomes such as BP, IQ, behavior and placental weight. An increased risk of intrauterine growth restriction and low Apgar scores was reported by one cohort study. However, the study designs used did not permit any conclusions to be drawn as to the relative importance of in-utero methyldopa exposure vs. the underlying effects of hypertension on the offspring. The ORs for perinatal mortality and preterm birth are reported as Forest plots in Supplementary Figs. 2 and 3.

### Diuretic exposure

One linkage cohort study [25] involving 47 386 offspring from Denmark and Scotland, reported the in-utero effects of diuretics. The study identified increased odds of preterm delivery associated with antenatal exposure to diuretics among Danish offspring (OR 1.8; 95% CI 1.2–2.7) but not Scottish offspring (OR 1.9; 95% CI 0.9–4.3). There was also an increased risk of low birth weight associated with diuretic use in the Danish offspring (OR 1.7; 95% CI 1.1–2.7) but not the Scottish offspring (OR 1.8; 95% CI 0.7–5.1).

### Studies assessing the long-term outcomes of antihypertensive exposure

One RCT [49] and three cohort studies [11,13,61] have investigated the effects of antihypertensive medication exposure *in utero* on later childhood development. The RCT [49] and two cohort studies [11,13] assessed labetalol vs. methyldopa and a control group, whereas the other cohort study [61] assessed the effect of clonidine. Table 6 reports details on study types, outcomes, and number of participants for studies assessing long-term effects of in-utero exposure to antihypertensive medication. Three studies were graded as mediocre [11,13,49] and one as poor [61] quality.

Cockburn *et al.*[49], in a mediocre RCT, investigated exposure to methyldopa in 195 offspring, followed up to 7 years of age. There were no differences in health outcomes, such as sight and hearing, IQ, and behavioural development between the methyldopa and untreated hypertensive groups.

Chan *et al.*[11], investigated the effects of in-utero exposure to labetalol or methyldopa in comparison with no potential teratogen exposure in 99 offspring 3–7 years postdelivery. The authors reported no significant differences in full-scale IQ, performance IQ or verbal IQ between the labetalol and the control groups in the mediocre cohort study.

In a mediocre cohort study of 202 patients, Pasker-De Jong *et al.*[13] investigated the potential effects of methyldopa or labetalol, compared with a hypertensive control group treated with bed rest. Labetalol exposure was associated with nonsignificantly higher odds of attention deficit hyperactive disorder compared with the methyldopa group (OR 2.3; 95% CI 0.7–7.3), and significantly higher odds compared with the bed rest group (OR 4.1; 95% CI 1.2–13.9). Although sleeping disorders were more frequent among the offspring of those treated with methyldopa compared with labetalol (OR 3.2; 95% CI 0.6–16.7) and bed rest (OR 4.5; 95% CI 0.9–23.2), neither result was statistically significant.

In a poor cohort study of 44 patients, Huisjes *et al.*[61] reported no significant difference in head circumference, neurological findings, or school performance between offspring exposed to clonidine and the unexposed groups. There was, however, an excess of hyperactivity and sleep disturbances reported by the parents and teachers in exposed offspring. Sleep disturbances were more severe in those children exposed to clonidine 300 μg or more *in utero*, suggesting a possible dose–response relationship.

## DISCUSSION

The current study reviewed 47 published studies reporting the effects of in-utero exposure to antihypertensive medication on the fetus and child. Thirty-two of these studies were of poor or mediocre quality, with small study populations, and incomplete adjustment for confounding, and lack of quality.

Although there is a widely held view that antihypertensive patients, such as beta-blockers, may be associated with a variety of detrimental fetal outcomes, such as low birth weight or congenital malformations, these beliefs are not based on robust data from appropriately designed and powered studies to conclusively confirm any associations. Furthermore, few studies have investigated the possible long-term outcomes following in-utero exposure to antihypertensive agents. The four studies which have done so have had small study populations, lacked statistical power and reported conflicting results. Although no IQ or developmental differences were reported for methyldopa and labetalol in two studies [11,49], sleep disturbance following clonidine [61] or methyldopa [13] exposure, and increased incidence of attention deficit hyperactivity disorder with labetalol [13] were reported in two of the four studies reporting long-term outcomes.

The five studies graded as excellent [29,31,33,51,57] suffered from confounding associated with the selection and nature of the comparison groups and the way in which medication exposure was reported.

One of the excellent studies assessing any antihypertensive medications by Lennestål *et al.*[29] reported an increased risk of cardiovascular defects following beta-blocker exposure, or exposure to more than one antihypertensive medication. This study, however relied on self-reported drug exposure during the first trimester by women during the first midwife visit. Analysis was based on women reporting any antihypertensive use (excluding beta-blockers), irrespective of whether they had a diagnostic code for chronic hypertension and women reporting beta-blocker use only if they also had a diagnostic code for chronic hypertension. Thus introducing recall bias and excluding women who may have been treated with beta-blockers but were not coded for chronic hypertension in their medical records.

The results of their study seem to indicate a beta-blocker driven association for congenital abnormalities; however, the increased frequency of such abnormalities may have been due to the underlying chronic hypertension. The authors fail to mention the severity of hypertension present in patients, which would impact on treatments used and outcomes of pregnancy. Furthermore, as the authors used only one point of reference to establish drug exposure, it is possible that as pregnancy progressed women may have experienced altered exposure to different antihypertensive patients or other potentially teratogenic agents, possibly confounding the results further. Lastly, because BP was not reported in the first midwife visit at which medication use was established it is not possible to determine whether patients in the untreated comparison group were truly normotensive. Therefore, the reported increase in the prevalence of congenital abnormalities and birth outcomes (preterm birth, placental abruption, delivery inductions) reported in this study may be related to underlying, or inadequately controlled, hypertension rather than antihypertensive therapy.

The second excellent study to assess any antihypertensive exposure during pregnancy by Nakhai-Pour *et al.*[31], reported an increased risk of being small for gestational age following exposure in the second or third trimester; however, there was no risk following exposure in the first trimester, or any increased risk of having a congenital anomaly following exposure to antihypertensive medication during any trimester in pregnancy. The authors did not disclose what the indication for or duration of the medication. Exposure therefore could be for a condition other than hypertension, or could be a one-time exposure, or for the last week of pregnancy, for example in the case of tocolysis. There was also no consideration of the effects of any underlying hypertension in the exposed group. The unexposed group may also have been affected by underlying hypertension as diagnosis of normotensive patients was not established.

The one study graded as excellent in quality assessing ACEi exposure during pregnancy, reported an increased risk of birth defects when compared with an untreated normotensive group, but no difference when compared with an untreated hypertensive control group [57]. However, the authors did not supply information on the type or severity of hypertension in women included in the untreated hypertensive comparison group. It is likely that women in the untreated hypertensive control group may have had less severe hypertension justifying no treatment, thus potentially confounding the results. The fact that the authors reported no differences in birth defects between the ACEi-treated and untreated hypertensive groups, and an increased risk for untreated hypertensive women in comparison with untreated normotensive women, suggests that hypertension alone, rather than medication, could be the association of the detrimental pregnancy outcomes.

The only study to assess calcium channel blocker exposure during the last 30 days of pregnancy rated as excellent, reported no increased risk of seizures compared with an unexposed normotensive comparison group [33]. This study was limited by the single outcome reported. The authors provided no information regarding the diagnosis, reason for calcium channel blocker therapy, other drugs taken, and no information regarding the unexposed cohort.

The fourth study deemed excellent was conducted by Orbach *et al.*[51], who investigated the outcomes following atenolol or methyldopa exposure during pregnancy. The authors reported an increased risk of preterm birth, being small for gestational age, low birth weight, and intrauterine growth restriction; however, all but low birth weight was also reported in the untreated hypertensive group. No increased risk of congenital defects or deaths was reported following exposure to atenolol or methyldopa. Those classed as having been exposed had a prescription for either atenolol or methyldopa in the first trimester. There was, however, no further information regarding concomitant drug exposure reported during the first trimester or the remainder of the pregnancy. The unexposed normotensive comparison group had no prescriptions for antihypertensive medication in the first and third trimester, and none had a diagnosis of chronic hypertension. However, it is possible that women in the untreated normotensive comparison group may have developed gestational hypertension. The last comparison group was untreated hypertensive women with a diagnosis of chronic hypertension but no prescriptions for any antihypertensive medication. The authors gave no information regarding the severity of hypertension in this control group possibly leading to selection bias as those treated may have had more severe hypertension.

Twelve of the 47 studies included in this review were RCTs; 11 of which were reported between 1982 and 2000. The gold standard for study design is an RCT, however, the need for randomization may conflict with the ethical treatment of hypertensive pregnant women and so introduce selection bias in the control groups.

Women included in these RCTs were often hospitalized for severe preeclampsia as reported by Heida *et al.*[38], which included a nontreated hypertensive group. The study populations included in the RCTs were therefore not representative of all hypertensive pregnant women and the use of treated and untreated preeclampsia introduces the possibility of further confounding. Furthermore, the fact that no RCTs have been undertaken since 2006 may again reflect the issues concerning the ethics of failure to treat pregnant women.

The study population size in the RCTs reviewed in this paper was between 25 and 300 patients. As the studies were small scale, they may have lacked statistical power.

The majority of reviewed studies were case–control or cohort studies in design, which inherently introduces the issue of confounding from unknown or unmeasured confounders, underlying or poorly controlled hypertension, and the issue of medication adherence. Studies using data from poison centers or teratology centers [22,24] may introduce selection bias, as women who contact these services and then are later enrolled onto a study are unlikely to be representative of the general population. In the six case–control studies [22,26–28,30,35], exposure was only reported by the women, and not validated by any other means, for example, prescription records, which may have introduced recall bias. Case–control studies also suffer from survival bias, as cases are identified and information gathered about exposure.

It is unclear whether the magnitude of the increased risks, such as birth defects [29,57], reported by these studies are clinically significant; however, even if these results are not deemed clinically significant to individuals, there may still be an important impact on populations which have a high prevalence of antihypertensive medication use. It is generally stated that the background incidence of congenital abnormalities in a healthy population is between 1 and 3%; however, this is likely to vary between populations. From this point of view, large-scale linkage studies have the advantage in permitting the calculation of a relatively accurate population based estimate of the outcomes of interest, compared with other types of methodology for collecting data.

The results are further confounded by the lack of information describing the types of hypertension treated in women included in the exposed groups. It is likely that the risk profiles and outcomes associated with chronic hypertension, gestational hypertension, and preeclampsia are different; therefore, a knowledge of the underlying condition is important and should be considered and reported. Only 16 of 47 studies reported in this review provided such information, and the majority of these studies assessed beta-blocker exposure.

Although it is recognized that the results of cohort studies should be reported as RR, ten of the cohort studies [13,24–26,29,33,40,43,51,57,65] used logistic regression analysis and reported results as OR values. Although the OR may represent a close approximation to the RR when the outcome of interest is rare (<10% of the general population/unexposed group), OR values may exaggerate the risk posed by the exposure studied and open the study to misinterpretation.

Treatment of hypertension during pregnancy and assessing the potential risks to the offspring is further complicated by the underlying disease. Untreated hypertension results in preterm birth, low birth weight and increased mortality. It would therefore be reasonable to suggest that poorly controlled hypertension may carry the same risks as untreated hypertension. Case–control and cohort studies reviewed in this paper have failed to determine whether hypertension was appropriately treated and controlled, and failed to assess adherence. Several studies reviewed [37,38,45,57] used an untreated hypertensive group to identify whether there was an increased risk in these cases; however, results were mixed with some studies reporting an increased risk compared with untreated hypertension [37,45], whereas others reported no difference [38,57].

Limitations to this review include exclusion of conference abstracts, unpublished studies, studies reported in any language other than English or German, which may have resulted in possible publication bias. However, when the literature search was performed, only two articles were excluded due to language. A further limitation was the wide date-range over which the included studies were published, the majority before 2000. As a result attempted contact with authors was either impractical or unsuccessful.

In conclusion, adverse child outcomes such as preterm birth, perinatal mortality, low birth weight, risk of congenital abnormalities, or other detrimental outcomes, following in-utero antihypertensive exposure have been reported in the literature. However, most published studies have had methodological weaknesses and/or lacked statistical power thus preventing any firm conclusions being drawn.

Further research in this area is required to ensure that health professionals have sufficient data to treat hypertension during pregnancy to firmly demonstrate a lack of detrimental outcomes.

## ACKNOWLEDGEMENTS

We acknowledge the support from the Farr Institute at Scotland. The Farr Institute at Scotland is supported by a 10-funder consortium: Arthritis Research UK, the British Heart Foundation, Cancer Research UK, the Economic and Social Research Council, the Engineering and Physical Sciences Research Council, the Medical Research Council, the National Institute of Health Research, the National Institute for Social Care and Health Research (Welsh Assembly Government), the Chief Scientist Office (Scottish Government Health Directorates), the Wellcome Trust, (MRC Grant No: MR/K007017/1).

### Conflicts of interest

There are no conflicts of interest.

## Supplementary Material

Supplemental Digital Content

## Figures and Tables

**TABLE 1:** Characteristics of studies Included in the systematic review that assess any antihypertensive exposure

Lead Author Year Reference	Arias 1979 [32]	Banhidy 2010 [26]	Caton 2008 [27]	Caton 2009 [28]	Lennestal 2009 [29]	Nakhai-Pour 2010a [31]	Nakhai-Pour 2010b [31]	Van Gelder 2015 [30]
Country	US	Hungary	US	US	Sweden	Canada	Canada	Netherlands
Study size	59	37 734	2887	9817	1063 238	56 334	59 033	12 821
Study design	CS	CC	CC	CC	CS	CC	CC	CC
Hypertension type	CH	CH/GH	NK	NK	NK	NK	NK	CH/GH/P
CASP Score (%)	67	53	76	76	87	94	94	82
Outcomes								
Preterm birth		Y			Y			
Small for gestational age	N				Y	Y		
Low birth weight	N	Y						
Head circumference								
Ponderal index								
Perinatal mortality								
Congenital defects		Y	Y	Y	Y		Y	Y
Seizures					Y			
Hypoglycaemia					Y			
IQ								
Sleep disorders								
Other long-term outcomes								
Fetal behavioral states								

Y denotes significant result reported for outcomes. N denotes no significant result reported for outcomes. CC, case–control study;CH, chronic hypertension; CS, cohort study; GH, gestational hypertension; IQ, intelligence quotient; NK, not known; P, preeclampsia; RCT, randomized controlled trial.

**TABLE 2:** Characteristics of studies Included in the systematic review that assess beta blocker exposure

Lead Author Year Reference	Bayliss 2002 [37]	Caton 2009 [28]	Chan 2010 [11]	Cooper 2006 [60]	Davis 2011 [12]	Fidler 1983 [52]	Gazzolo 1998 [55]	Heida 2012 [38]
Country	UK	US	Canada	US	US	UK	Italy	Netherlands
Study size	491	9817	99	29 507	87 407	100	117	95
Study design	CS	CC	CS	CS	CS	RCT	CS	CS
Hypertension type	CH	NK	NK	NK	NK	NK	GH	P
CASP Score (%)	53	76	76	73	73	63	40	53
Outcomes								
Preterm birth								N
Small for gestational age	Y							N
Low birth weight	Y					N		N
Head circumference						N		
Ponderal index	Y	Y						
Perinatal mortality								Y
Congenital defects				Y	Y			
Seizures					Y			
Hypoglycaemia					Y			
IQ			N					
Sleep disorders								
Other Long-term outcomes								
Fetal behavioral states							Y	

Lead Author Year Reference	Lennestal 2009 [29]	Lieberman 1978 [46]	Lip 1997 [39]	Lydakis 1999 [45]	Macpherson 1986 [48]	Meidahl 2012 [40]	Munshi 1992 [41]	Orbach 2013 [51]
Country	Sweden	UK	UK	UK	UK	Denmark	India	Israel
Study size	1063 238	23	398	312	22	911 685	129	100 029
Study design	CS	CS	CS	CS	CS	CS	CS	CS
Hypertension type	NK	NK	NK	NK	NK	NK	GH	NK*
CASP Score	87	47	47	73	60	73	33	87
Outcomes								
Preterm birth	Y			Y		Y		Y
Small for gestational age	Y			Y		Y		Y
Low birth weight		Y	Y	Y				Y
Head circumference							N	Y
Ponderal index			Y					Y
Perinatal mortality						Y		N
Congenital defects	Y							N
Seizures	Y							
Hypoglycaemia	Y						Y	
IQ								
Sleep disorders								
Other long-term outcomes								
Other immediate outcomes					N			

Lead Author Year Reference	Pickles 1992 [42]	Plouin 1988 [53]	Ray 2001 [43]	Rubin 1983 [44]	Sibai 1987 [47]	Sibai 1990 [54]	Vigil-DeGracia 2006 [64]	Xie 2014 [65]
Country	UK	France	Canada	UK	USA	USA	Panama	Canada
Study size	144	176	1948	120	200	263	200	1223
Study design	RCT	RCT	CS	RCT	RCT	RCT	RCT	CS
Hypertension type	GH	NK	CH	GH	P	CH	NK	CH/GH
CASP Score (%)	63	88	60	75	75	81	81	47
Outcomes								
Preterm birth			Y			N		N
Small for gestational age	N		Y		Y	N		
Low birth weight					N	N	N	
Head circumference						N		
Ponderal index								
Perinatal mortality			N				N	N
Congenital defects								
Seizures								Y
Hypoglycaemia		N		N		N	N	
IQ								Y
Sleep disorders								
Other long-term outcomes								
Fetal behavioral states								

Y denotes significant result reported for outcomes. N denotes no significant result reported for outcomes. CC, case–control study; CH, chronic hypertension; CS, cohort study; GH, gestational hypertension; IQ, intelligence quotient; NK*, not known for the treated group, however the untreated hypertensive group had CH; NK, not known; P, preeclampsia; RCT, randomized controlled trial.

**TABLE 3:** Characteristics for studies included in the systematic review that assess calcium channel blocker exposure

Lead Author Year Reference	Bateman 2015 [33]	Davis 2011 [12]	Fenakel 1991 [63]	Gruppo 1998 [34]	Hall 2000 [62]	Magee 1996 [23]	Sorensen 2001 [35]	Weber-Schoendorfer 2008 [24]
Country	US	US	Israel	Italy	S. Africa	Canada; US; UK	Hungary	Germany; France; the Netherlands; Israel; Spain; Italy; Finland
Study size	2529 636	87 407	49	283	150	156	54 016	1105
Study design	CS	CS	RCT	RCT	RCT	CS	CC	CS
Hypertension type	NK	NK	P	NK	NK	NK	NK	NK
CASP Score (%)	87	73	63	63	75	40	76	47
Outcomes								
Preterm birth						Y		Y
Small for gestational age						N		
Low birth weight			N	N				Y
Head circumference								
Ponderal index								
Perinatal mortality			N	N	Y			Y
Congenital defects		Y	N			N	Y	N
Seizures	N	Y						
Hypoglycaemia		Y						
IQ								
Sleep disorders								
Other long-term outcomes								
Fetal behavioral states								

Y denotes significant result reported for outcomes. N denotes no significant result reported for outcomes. CC, case–control study; CH, chronic hypertension; CS, cohort study; GH, gestational hypertension; IQ, intelligence quotient; NK, not known; P, preeclampsia; RCT, randomized controlled trial.

**TABLE 4:** Characteristics for studies Included in the systematic review that assess angiotensin-converting enzyme inhibitor and angiotensin receptor blocker exposure

Lead Author Year Reference	Caton 2009 [28]	Cooper 2006 [60]	Diav-Citrin 2011 [22]	Karthikeyan 2011 [56]	Lennestal 2009 [29]	Li 2011 [57]	Moretti 2011 [58]	Tabacova 2003 [59]
Country	US	US	Israel/Italy	UK	Sweden	US	Canada	US
Study size	9817	29 507	1003	94	1063 238	465 754	388	108
Study design	CC	CS	CS	CS	CS	CS	CS	CS
Hypertension type	NK	NK	NK	NK	NK	NK	NK	NK
CASP Score (%)	76	73	47	33	87	93	53	33
Outcomes								
Preterm birth			Y		Y		Y	Y
Small for gestational age			Y		Y			
Low birth weight							Y	
Head circumference								
Ponderal index	Y							
Perinatal mortality			Y	N			Y	Y
Congenital defects		Y	N	N	Y	Y	N	Y
Seizures					Y			
Hypoglycaemia					Y			
IQ								
Sleep disorders								
Other long-term outcomes								
Fetal behavioral states								

Y denotes significant result reported for outcomes. N denotes no significant result reported for outcomes. CC, case–control study; CH, chronic hypertension; CS, cohort study; GH, gestational hypertension; IQ, intelligence quotient; NK, not known; P, preeclampsia; RCT, randomized controlled trial.

**TABLE 5:** Characteristics for studies Included in the Systematic Review that assess methyldopa exposure

Lead Author Year Reference	Cockburn 1982 [49]	Fidler 1983 [52]	Orbach 2013 [51]	Weitz 1987 [50]	Xie 2014 [65]
Country	UK	UK	Israel	USA	Canada
Study size	195	100	100 029	25	1223
Study design	RCT	RCT	CS	RCT	CS
Hypertension type	NK	NK	NK*	CH	CH/GH
CASP Score (%)	69	63	87	75	47
Outcomes					
Preterm birth			Y		N
Small for gestational age			Y		
Low birth weight		N	Y	N	
Head circumference	Y	N	Y	N	
Ponderal index			Y		
Perinatal mortality			N		N
Congenital defects			N		
Seizures					Y
Hypoglycaemia					
IQ	N				Y
Sleep disorders					
Other long-term outcomes	N				
Fetal behavioral states					

Y denotes significant result reported for outcomes. N denotes no significant result reported for outcomes. CC, case–control study; CH, chronic hypertension; CS, cohort study; GH, gestational hypertension; IQ, intelligence quotient; NK*, Not known for the treated group, however the untreated hypertensive group had CH; NK, not known; P, preeclampsia; RCT, randomized controlled trial.

**TABLE 6:** Characteristics for studies included in the systematic review that assess long-term outcomes following in-utero exposure to antihypertensive medication

Lead Author Year Reference	Chan 2010 [11]	Cockburn 1982 [49]	Huisjes 1986 [61]	Pasker-De Jong 2010 [13]
Country	Canada	UK	Netherlands	Netherlands
Study size	99	195	44	202
Study design	CS	RCT	CS	CS
Hypertension type	NK	NK	NK	GH
CASP Score (%)	67	69	47	67
Outcomes				
Preterm birth				
Small for gestational age				
Low birth weight				
Head circumference		Y	N	
Ponderal index				
Perinatal mortality				
Congenital defects				
Seizures				
Hypoglycaemia				
IQ	N	N		
Sleep disorders			Y	Y
Other long-term outcomes		N	N	Y
Fetal behavioral states				

Y denotes significant result reported for outcomes. N denotes no significant result reported for outcomes. CC, case–control study; CH, chronic hypertension; CS, cohort study; GH, gestational hypertension; IQ, intelligence quotient; NK, not known; P, preeclampsia; RCT, randomized controlled trial.

## Reviewers’ Summary Evaluations

### Reviewer 1

Strengths: A better understanding of the impact of in utero exposure to antihypertensive medication is important, particularly with the increasing prevalence of hypertension and similar risk factors in women of childbearing age. This manuscript nicely brings together the current state of the literature. In doing so, the need for more high quality, well designed studies on potential benefits and adverse outcomes of antihypertensive treatments in pregnancy becomes apparent.

Weaknesses: The field provides insufficient information to discriminate between potentially complex adverse effects of the different types of hypertensive conditions (preeclampsia, pregnancy-induced or preexisting hypertension) and the antihypertensive medications used to treat them.

### Reviewer 2

This article's strengths are its use of consensus-determined systematic review methods, careful consideration of study quality, and inclusion of relevant details from many different study types. This article's weaknesses are the inconclusive nature of the results, which was dependent on the studies available for review.
