# Supplementary material for: In-utero exposure to antihypertensive medication and neonatal and child health outcomes: a systematic review
Source: J Hypertens. 2017 Jul 18;35(11):2123–37. doi: 10.1097/HJH.0000000000001456 (PMC5625961; doi:10.1097/HJH.0000000000001456)
Supplement: Supplemental Digital Content [file jhype-35-2123-s001.doc]

**Figure 1 (Supplemental file 1):** PRISMA flow diagram**Figure 2 (Supplemental file 2): Forest plot of Odds Ratio (OR) for preterm delivery, following *in utero* exposure to anti-hypertensive agents. BB= Beta blocker; CCB = Calcium channel blocker.**

Records identified through database searching
(n = 809)

Additional records identified through other sources
(n = 72)

**Screening**

Records after duplicates removed
(n = 688)

**Eligibility**

**Included**

**Identification**

Records screened
(n = 688)

Records excluded
(n = 644)

Full-text articles assessed for eligibility
(n = 54)

Full-text articles excluded, with reasons (n = 7)

No child info included in results = 2;

Same info already reported = 2;

Unable to source full text = 1;

Conference abstract with no further info = 1; Dissertation with no full text = 1

Studies included in qualitative synthesis
(n = 47)


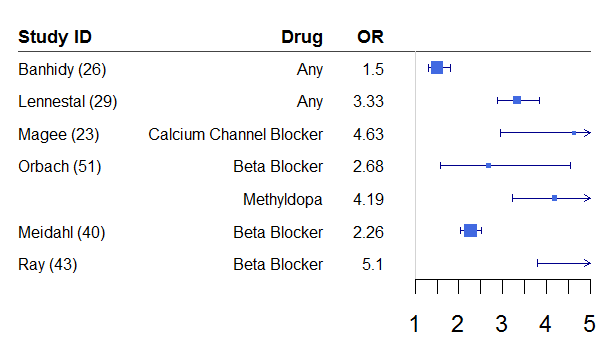


**Supplemental Figure 2**. Forest plot of Odds Ratio (OR) for preterm delivery, following *in utero* exposure to anti-hypertensive agents. BB= Beta blocker; CCB = Calcium channel blocker.


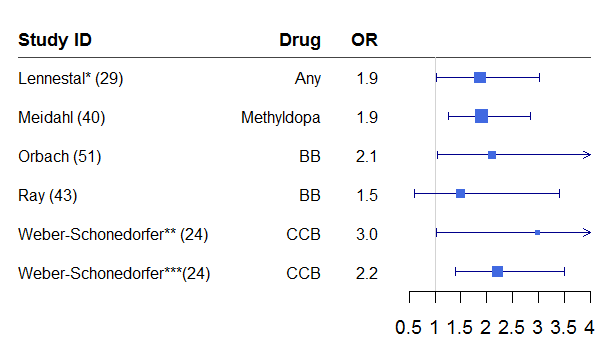


**Supplemental Figure 3.** Forest plot of Odds Ratios (OR) for perinatal mortality following *in utero* exposure to anti-hypertensive agents. *RR reported; **Stillbirths; ***Miscarriages; BB = Beta blocker; CCB = Calcium channel blocker


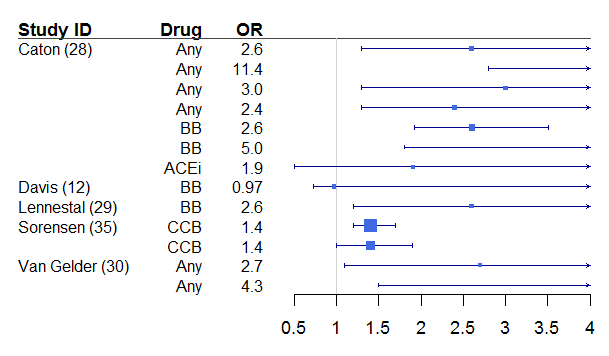


**Supplemental Figure 4.** Forest plot of Odds Ratios (OR) for presence of congenital cardiovascular defects following *in utero* exposure to anti-hypertensive agents. BB = Beta blocker; CCB = Calcium channel blocker

**Table 7 (Supplemental file 5):** Modified CASP for Case Control Studies. Y denotes an appropriate level of reporting in the section specified. N denotes insufficient information reported.

| Lead Author  Year  References | Caton  2008  (27) | Caton  2009  (28) | Nakhai-Pour 2010a  (31) | Nakhai-Pour 2010b  (31) | Sorensen  2001  (35) | Van Gelder  2015  (30) |
| --- | --- | --- | --- | --- | --- | --- |
| Study period | 1997- 2002 | 1997-2003 | ? | ? | 1980-1996 | 1998-2010 |
| Study population representative | Y | Y | Y | Y | Y | Y |
| Population described | Y | Y | Y | Y | Y | Y |
| Cases recruited fairly | Y | Y | Y | Y | Y | Y |
| Healthy Control | Y | Y | Y | Y | Y | Y |
| Untreated Disease control | N | N | N | N | N | N |
| Cases and controls recruited from same population | Y | Y | Y | Y | Y | Y |
| Cases and controls recruited in same way | Y | Y | Y | Y | Y | Y |
| Inclusion and exclusion criteria stated for cases | Y | Y | Y | Y | Y | Y |
| Inclusion and exclusion criteria stated for controls | Y | Y | Y | Y | N | Y |
| Cases validated | Y | Y | Y | Y | Y | Y |
| Exposure validated | N | N | Y | Y | ? | N |
| Data linkage | N | N | Y | Y | Y | N |
| Appropriate confounders accounted for | Y | Y | Y | Y | Y | Y |
| Statistics appropriate | Y | Y | Y | Y | Y | Y |
| Outcomes measured objectively | Y | Y | Y | Y | Y | Y |
| Response rates equal between groups | ? | N | Y | Y | ? | Y |
| Follow up sufficient | Y | Y | Y | Y | Y | Y |
| Total out of 17 | 13 | 13 | 16 | 16 | 13 | 14 |

**Table 8 (Supplemental file 6):** Modified CASP for Cohort Studies. Y denotes an appropriate level of reporting in the section specified. N denotes insufficient information reported.

| Lead Author  Year  References | Arias 1979  (32) | Banhidy 2010  (26) | Bateman 2015  (33) | Bayliss 2002  (37) | Chan  2010  (11) | Cooper 2006  (60) | Davis  2011  (12) | Diav-Citrin 2011  (22) | Gazzolo  1998  (55) |
| --- | --- | --- | --- | --- | --- | --- | --- | --- | --- |
| Study period | ? | 1980- 1996 | 2000- 2007 | 1980- 1999 | 1997-2002 | 1985-2000 | 1996-2000 | 1994- 2008 | 1989-1993 |
| Prospective or retrospective? | P | R | P | R | P | R | R | P | P |
| Study population representative | N | Y | Y | N | N | Y | Y | N | N |
| Population described | Y | N | Y | N | Y | N | Y | Y | N |
| Healthy Comparator | N | Y | Y | Y | Y | Y | Y | Y | Y |
| Untreated Disease Comparator | Y | N | N | Y | N | N | N | N | N |
| Cases and comparators recruited from same population | Y | Y | Y | N | Y | Y | Y | Y | N |
| Cases and comparators recruited in same way | N | Y | Y | N | Y | N | Y | Y | N |
| Inclusion and exclusion criteria stated for cases | Y | Y | Y | Y | Y | Y | N | N | N |
| Inclusion and exclusion criteria stated for comparators | Y | N | Y | N | Y | Y | N | N | N |
| Exposure validated | Y | N | Y | Y | N | N | Y | N | Y |
| Data linkage | N | Y | Y | N | N | Y | Y | N | N |
| Outcome validated | Y | Y | Y | Y | Y | Y | Y | N | Y |
| Confounders accounted for | N | N | Y | N | N | Y | N | N | N |
| Statistics appropriate | Y | N | N | Y | Y | Y | Y | Y | Y |
| Outcomes measured objectively | Y | Y | Y | Y | Y | Y | Y | Y | Y |
| Follow up time sufficient | Y | Y | Y | Y | Y | Y | Y | Y | Y |
| Total out of 15 | 10 | 9 | 13 | 8 | 10 | 11 | 11 | 7 | 6 |

| Lead Author  Year  References | Heida  2012  (38) | Huisjes  1986  (61) | Karthikeyan 2011  (56) | Lennestal  2009  (29) | Li  2011  (57) | Lieberman 1978  (46) | Lip  1997  (39) |
| --- | --- | --- | --- | --- | --- | --- | --- |
| Study period | 2003-2008 | 1974- 1981 | - | 1995-2006 | 1995-2008 | 1970- 1973 | 1980-1995 |
| Prospective or retrospective? | R | R | P | R | R | R | P |
| Study population representative | N | N | N | Y | Y | N | N |
| Population described | Y | N | Y | Y | Y | Y | N |
| Healthy Comparator | N | N | N | Y | Y | N | Y |
| Untreated Disease Comparator | Y | Y | N | N | Y | Y | Y |
| Cases and comparators recruited from same population | N | N | N | Y | Y | N | N |
| Cases and comparators recruited in same way | N | Y | N | Y | Y | N | N |
| Inclusion and exclusion criteria stated for cases | Y | N | N | Y | Y | N | N |
| Inclusion and exclusion criteria stated for comparators | N | N | N | Y | Y | N | N |
| Exposure validated | Y | Y | Y | Y | Y | Y | Y |
| Data linkage | N | N | N | Y | Y | N | N |
| Outcome validated | Y | Y | Y | Y | Y | Y | Y |
| Confounders accounted for | N | N | N | Y | Y | N | Y |
| Statistics appropriate | Y | Y | N | N | N | Y | Y |
| Outcomes measured objectively | Y | Y | Y | Y | Y | Y | Y |
| Follow up time sufficient | Y | Y | Y | Y | Y | Y | N |
| Total out of 15 | 8 | 7 | 5 | 13 | 14 | 7 | 7 |

| Lead Author  Year  References | Lydakis  1999  (45) | MacPherson 1986  (48) | Magee  1996  (23) | Meidahl Petersen 2012  (40) | Moretti 2011  (58) | Munshi 1992  (41) | Olesen  2001  (25) |
| --- | --- | --- | --- | --- | --- | --- | --- |
| Study period | 1980-1997 | ? | 1984-1994 | 1995-2008 | ? | 1989- 1991 | 1991- 1998 |
| Prospective or retrospective? | R | P | P | R | P | N | N |
| Study population representative | N | N | N | Y | N | N | Y |
| Population described | Y | Y | Y | N | Y | N | N |
| Healthy Comparator | N | Y | Y | Y | Y | Y | Y |
| Untreated Disease Comparator | Y | N | N | N | Y | Y | N |
| Cases and comparators recruited from same population | Y | Y | Y | Y | Y | N | Y |
| Cases and comparators recruited in same way | Y | Y | Y | Y | Y | N | Y |
| Inclusion and exclusion criteria stated for cases | Y | N | N | Y | N | N | N |
| Inclusion and exclusion criteria stated for comparators | N | N | N | N | N | N | N |
| Exposure validated | Y | Y | N | Y | N | N | Y |
| Data linkage | N | N | N | Y | N | N | Y |
| Outcome validated | Y | Y | N | Y | N | N | Y |
| Confounders accounted for | Y | N | N | Y | N | N | Y |
| Statistics appropriate | Y | Y | Y | N | Y | Y | Y |
| Outcomes measured objectively | Y | Y | Y | Y | Y | Y | Y |
| Follow up time sufficient | Y | Y | N | Y | Y | Y | Y |
| Total out of 15 | 11 | 9 | 6 | 11 | 8 | 5 | 11 |

| Lead Author  Year  References | Orbach  2013  (51) | Pasker de Jong 2010  (13) | Ray  2001  (43) | Tabacova  2003  (59) | Weber-Schoendorfer 2008  (24) | Xie  2014  (65) |
| --- | --- | --- | --- | --- | --- | --- |
| Study period | 1998-2008 | 1983-1987 | 1986-1995 | 1986-2000 | 1986-2003 | 1990-2005 |
| Prospective or retrospective? | R | R | P | R | R | P |
| Study population representative | Y | N | Y | N | N | N |
| Population described | Y | Y | N | N | Y | Y |
| Healthy Comparator | Y | N | Y | N | Y | N |
| Untreated Disease Comparator | Y | Y | N | N | N | N |
| Cases and comparators recruited from same population | Y | Y | Y | N | Y | N |
| Cases and comparators recruited in same way | Y | Y | Y | N | Y | N |
| Inclusion and exclusion criteria stated for cases | Y | Y | N | Y | N | Y |
| Inclusion and exclusion criteria stated for comparators | N | N | N | N | N | N |
| Exposure validated | Y | Y | Y | N | N | Y |
| Data linkage | Y | N | N | N | N | N |
| Outcome validated | Y | Y | Y | Y | N | Y |
| Confounders accounted for | Y | Y | Y | N | Y | Y |
| Statistics appropriate | N | N | N | Y | N | N |
| Outcomes measured objectively | Y | Y | Y | Y | Y | Y |
| Follow up time sufficient | Y | Y | Y | Y | Y | Y |
| Total out of 15 | 13 | 10 | 9 | 5 | 7 | 7 |

**Table 9 (Supplemental file 7):** Modified CASP for Randomised Controlled Trials. Y denotes an appropriate level of reporting in the section specified. N denotes insufficient information reported.

| Lead Author  Year  References | Cockburn  1982  (49) | Fenakel  1991  (63) | Fidler  1983  (52) | Gruppo  1998  (34) | Hall  2000  (62) | Pickles  1992  (42) |
| --- | --- | --- | --- | --- | --- | --- |
| Study period | 1970-? | 1985- 1988 | ? | 1992-1994 | 1994-1997 | ? |
| Study setting appropriate | Y | Y | Y | Y | Y | Y |
| Population described | Y | Y | Y | Y | Y | Y |
| Intervention and control described | Y | Y | Y | Y | Y | Y |
| Appropriate control | Y | N | Y | Y | N | Y |
| Randomisation described | N | N | N | Y | Y | N |
| Blinding described | N | N | N | N | Y | N |
| Appropriate blinding | N | N | N | N | Y | N |
| Both groups treated the same | Y | Y | Y | N | N | Y |
| Intention to treat | N | N | N | Y | N | N |
| Direct stat analysis | Y | Y | Y | Y | Y | Y |
| Outcomes validated | Y | Y | Y | Y | Y | Y |
| Clinically relevant outcomes | Y | Y | Y | Y | Y | Y |
| Follow up sufficient | Y | Y | Y | Y | Y | Y |
| All patients accounted for | N | Y | Y | N | Y | Y |
| Were all clinically important outcomes considered | Y | N | N | N | N | N |
| Can the results be applied to the general population | Y | Y | N | N | Y | N |
| Total out of 16 | 11 | 10 | 10 | 10 | 12 | 10 |

| Lead Author  Year  References | Plouin  1988  (53) | Rubin  1983  (44) | Sibai  1987  (47) | Sibai  1990  (54) | Vigil-De Gracia  2006  (64) | Weitz  1987  (50) |
| --- | --- | --- | --- | --- | --- | --- |
| Study period | 1983- 1985 | ? | ? | ? | 2003- 2004 | ? |
| Study setting appropriate | Y | Y | Y | Y | Y | Y |
| Population described | Y | Y | Y | Y | Y | Y |
| Intervention and control described | Y | Y | Y | Y | Y | Y |
| Appropriate control | Y | Y | Y | Y | N | Y |
| Randomisation described | Y | N | Y | Y | Y | N |
| Blinding described | N | N | N | N | N | Y |
| Appropriate blinding | N | Y | N | N | N | N |
| Both groups treated the same | Y | Y | Y | Y | Y | Y |
| Intention to treat | Y | N | N | Y | Y | N |
| Direct stat analysis | Y | Y | Y | Y | Y | Y |
| Outcomes validated | Y | Y | Y | Y | Y | Y |
| Clinically relevant outcomes | Y | Y | Y | Y | Y | Y |
| Follow up sufficient | Y | Y | Y | Y | Y | Y |
| All patients accounted for | Y | Y | Y | Y | Y | Y |
| Were all clinically important outcomes considered | Y | N | N | N | Y | N |
| Can the results be applied to the general population | Y | Y | Y | Y | Y | Y |
| Total out of 16 | 14 | 12 | 12 | 13 | 13 | 12 |
